# Risk factors, severity-related factors, and clinical outcomes of immune checkpoint inhibitor-associated pneumonitis in elderly patients with lung cancer

**DOI:** 10.3389/fonc.2026.1944973

**Published:** 2026-09-03

**Authors:** Ying Hu, Shuhua Chen, Yingying Lu, Lingjuan Yang

**Affiliations:** Department of Oncology, The Second Affiliated Hospital of Naval Medical University, Shanghai, China

**Keywords:** checkpoint inhibitor pneumonitis, elderly patients, immune checkpoint inhibitors, lung cancer, risk factors

## Abstract

**Objective:**

This study aimed to investigate the clinical characteristics, risk factors, severity-related factors, and clinical outcomes of immune checkpoint inhibitor-associated pneumonitis (CIP) after immune checkpoint inhibitor (ICI) treatment in elderly patients with lung cancer, providing evidence for risk identification and nursing management during ICI therapy.

**Methods:**

Clinical data from 206 elderly patients with lung cancer who received ICI therapy at the Second Affiliated Hospital of Naval Medical University between March 2024 and February 2026 were retrospectively collected. The incidence, onset time, and Common Terminology Criteria for Adverse Events (CTCAE) grade of CIP were recorded. Univariate and multivariable logistic regression analyses were performed to identify risk factors for CIP. Patients with CIP were classified into mild (grades 1–2) and moderate-to-severe (grade ≥3) groups. Clinical characteristics and outcomes were compared between the two groups. Firth penalized logistic regression was used to identify factors associated with CIP severity.

**Results:**

Among the 206 patients, 33 developed CIP (16.0%). The median time to CIP onset was 58 (45–82) days, and 81.8% of cases occurred within 90 days after ICI initiation. Twenty-two patients (66.7%) had mild CIP and 11 (33.3%) had moderate-to-severe CIP. Multivariable logistic regression identified prior thoracic radiotherapy (OR = 3.34, 95% CI: 1.45–7.69), pulmonary comorbidity (OR = 2.94, 95% CI: 1.30–6.63), and combination therapy (OR = 3.68, 95% CI: 1.36–10.00) as independent risk factors for CIP. Firth penalized logistic regression showed that pulmonary comorbidity (OR = 8.44, 95% CI: 1.42–95.17, P = 0.017) and prior thoracic radiotherapy (OR = 5.56, 95% CI: 1.07–39.63, P = 0.041) were associated with moderate-to-severe CIP.

**Conclusion:**

Most CIP cases occurred within the first 3 months after ICI treatment and were mild. Prior thoracic radiotherapy, pulmonary comorbidity, and combination therapy increased the risk of CIP, whereas pulmonary comorbidity and prior thoracic radiotherapy were associated with moderate-to-severe CIP in exploratory analysis. The above findings may provide a reference for the identification of patients with relevant risk factors, early monitoring, and nursing management during ICI treatment.

## 1 Introduction

ICIs are widely used to treat advanced lung cancer. They can improve survival outcomes in selected patients. Evidence from clinical trials supports the survival benefit of ICIs in advanced lung cancer. First-line pembrolizumab extended overall survival in patients with advanced non-small cell lung cancer (NSCLC) with high expression of programmed death-ligand 1 (PD-L1) in the KEYNOTE-024 study; the 5-year overall survival rate was almost twice that of chemotherapy alone (1). Similarly, the PACIFIC trial reported a 5-year overall survival rate of 42.9% in patients with unresectable stage III NSCLC who received durvalumab as consolidation therapy (2). However, with the wide use of ICIs, immune-related adverse events (irAEs) have become a key issue affecting treatment safety and continuity. One common immune-related adverse event that affects the lungs is CIP. Its incidence is about 3%–6% in clinical trials and 5%–19% in real-world studies of patients with lung cancer (3). Although its incidence is relatively low, CIP may cause severe lung injury. Although its incidence is relatively low, it can cause severe lung injury and is one of the immune-related adverse events that need to be paid attention to during ICI treatment (4). Previous studies have pointed out that although ICI-related pneumonia is rare, it may become an important cause of ICI-related death (4, 5).

Elderly patients with lung cancer may have a further increased risk of CIP due to reduced lung function reserve, higher prevalence of underlying lung diseases, and often receive comprehensive treatments such as radiotherapy and chemotherapy (6). The underlying mechanism centers on loss of immune tolerance in the lung: ICIs drive excessive T-cell activation and pro-inflammatory cytokine release, tipping local pulmonary immune homeostasis toward inflammatory injury (7, 8). The lung tissue of elderly patients and those with underlying lung diseases are more susceptible to such immune-inflammatory damage. However, current research on risk factors for CIP mainly focuses on the general lung cancer population, and the evidence for the special group of elderly patients is still limited. Previous studies have reported that underlying pulmonary disease, a prior thoracic radiotherapy, and combination therapy may be associated with the occurrence of CIP. However, most studies have focused on the general lung cancer population, and the characteristics of CIP occurrence, factors associated with severity, and clinical outcomes in elderly patients remain insufficiently described (9, 10). Elderly patients often have reduced pulmonary reserve, multiple comorbidities, and complex treatment needs. Therefore, further evaluation of CIP risk characteristics and disease burden in this population has important clinical significance. From the perspective of nursing practice, identifying only whether CIP occurs is insufficient to meet clinical management needs. More importantly, identifying risk factors and related clinical characteristics associated with moderate-to-severe CIP and conducting targeted monitoring and early assessment based on these risk factors may facilitate early recognition and standardized management of CIP.

Based on this background, this study retrospectively analyzed the characteristics of CIP occurrence during ICI treatment in elderly patients with lung cancer, investigated factors associated with CIP occurrence and severity, and further evaluated the clinical management and outcomes of patients with different CIP severity levels. The findings provide a reference for risk factor identification, early monitoring, and nursing management during ICI treatment in elderly patients with lung cancer.

## 2 Materials and methods

### 2.1 Study population

This single-center retrospective study included elderly patients with lung cancer who received ICI treatment in the Department of Oncology at the Second Affiliated Hospital of Naval Medical University between March 2024 and February 2026. Clinical information was obtained from the electronic medical record system. Patients were eligible if they met the following criteria: (1) aged 65 years or older; (2) histopathological or cytological confirmation of lung cancer in accordance with the Guidelines for Diagnosis and Treatment of Primary Lung Cancer in China (11); (3) Patients with evaluable active lesions at the initiation of ICI treatment were included if they met one of the following criteria: (a) newly diagnosed stage III–IV lung cancer receiving ICI therapy; or (b) local recurrence or distant metastasis after previous surgery, radiotherapy, or other anticancer treatments, followed by ICI therapy; (4) receipt of at least one cycle of programmed death-1 (PD-1) or PD-L1 inhibitor therapy; and (5) comprehensive clinical and follow-up information. The following patients were excluded: (1) active pulmonary infection or interstitial pneumonia due to other causes before ICI treatment; (2) patients who were receiving drugs with known pulmonary toxicity before ICI treatment, such as bleomycin or amiodarone; (3) patients with other active malignancies requiring anticancer treatment, or severe systemic diseases that could affect ICI treatment regimens, CIP diagnosis, or clinical outcome evaluation; (4) patients receiving neoadjuvant ICI therapy; (5) incomplete clinical or follow-up records. A total of 206 eligible patients were included. This study was approved by the ethics committee of our hospital. Since the study employed anonymized data that was gathered after the fact, informed consent was not required.

### 2.2 Data collection

All data were extracted from the hospital electronic medical record system by two investigators independently, with discrepancies resolved by a third reviewer. Demographic and baseline clinical variables collected included age, sex, body mass index (BMI), smoking history, and Eastern Cooperative Oncology Group (ECOG) performance status. Disease-related data included pathological type of lung cancer, clinical stage determined according to the 8th edition of the American Joint Committee on Cancer/Union for International Cancer Control (AJCC/UICC) TNM staging system, and pulmonary comorbidity. Pulmonary comorbidity was defined as the presence of one or more of the following chronic, stable-phase conditions documented before ICI treatment: chronic obstructive pulmonary disease, emphysema, pulmonary fibrosis, interstitial lung disease, previous pulmonary tuberculosis, or other chronic structural lung diseases; patients could have more than one component condition simultaneously. This differs from the exclusion criterion of active pulmonary infection or interstitial pneumonia due to other causes (see Study population), which referred to acute or active inflammatory processes present at the time of ICI initiation, rather than pre-existing chronic structural lung disease. The distribution of individual component conditions among patients with pulmonary comorbidity is presented in **Supplementary Table 1**.

Treatment-related data included ICI type (PD-1/PD-L1 inhibitor), prior thoracic radiotherapy (defined as thoracic radiotherapy completed before ICI initiation), combination radiotherapy (defined as thoracic radiotherapy administered concurrently with or after ICI initiation, i.e., during the course of ICI treatment), combination chemotherapy (defined as chemotherapy administered concurrently with or after ICI initiation, i.e., during the course of ICI treatment), and number of ICI treatment cycles. In this cohort, combination chemotherapy and radiotherapy were part of the initial planned treatment regimen; no patient received new chemotherapy or radiotherapy after CIP diagnosis. Outcome data included the occurrence of CIP, time from ICI initiation to CIP onset, CTCAE grade, corticosteroid use, ICI management (temporary interruption, no discontinuation, or permanent discontinuation), length of hospital stay, and clinical outcome of CIP.

### 2.3 Definition of CIP and follow-up

CIP was based on clinical manifestations, chest computed tomography (CT) findings, and exclusion diagnosis. For cases with complex or uncertain diagnoses, the final assessment was based on the evaluation of chest CT imaging features by radiologists and the opinions of oncologists and respiratory specialists. Symptomatically, CIP was suspected when a patient developed a new or worsening cough, breathlessness, chest tightness, fever, falling exercise capacity, or desaturation. On chest CT, the corresponding signs were fresh or expanding ground-glass opacities, patchy infiltrates, areas of consolidation, or interstitial change. CIP was diagnosed after excluding bacterial, viral, or fungal pneumonia, tumor progression, radiation pneumonitis, and other drug-related lung injuries. Bronchoscopy and bronchoalveolar lavage were not routinely performed in all patients but were selectively conducted when further evaluation was required to exclude infection or other pulmonary diseases. In patients with prior thoracic radiotherapy, radiation pneumonitis was differentiated from CIP based on the radiotherapy history, radiation field, and imaging distribution patterns. The severity of CIP was classified using the Common Terminology Criteria for Adverse Events version 5.0 (CTCAE 5.0). Patients with CIP were divided into a mild group and a moderate-to-severe group. CTCAE grades 1–2 were defined as mild CIP, and CTCAE grade ≥3 was defined as moderate-to-severe CIP. The diagnosis and grading of CIP followed relevant guidelines for the management of immune-related adverse events (12).

Follow-up started on the date of the first ICI treatment and ended at the last follow-up visit (June 30, 2026), death, or data lock, whichever occurred first. Follow-up data included CIP occurrence, onset time, severity, treatment management, ICI interruption, and clinical outcome. The median follow-up duration of the whole cohort was 499 days (about 16.4 months), and 92.7% (191/206) of patients had a follow-up duration of at least 6 months. According to prior research, the majority of CIP cases happened within six months following treatment, with a median onset time of between 2.8–4.6 months (9). Therefore, the follow-up duration in this study was longer than the main high-risk window, which helped provide relatively sufficient observation of CIP events.

Clinical outcome of CIP was assessed at the last follow-up visit or at data lock (June 30, 2026), whichever occurred first, and was reported using two separate variables. CIP trajectory was classified as: (1) improved: complete or partial resolution of CIP-related symptoms and radiological abnormalities; (2) stable: no significant change in CIP-related symptoms or imaging from the time of diagnosis; (3) progression: persistent worsening of CIP-related respiratory symptoms or imaging findings despite treatment. All-cause death during follow-up was reported as a separate binary variable, irrespective of CIP trajectory at the time of death, because cause of death could not be reliably adjudicated in this retrospective cohort.

### 2.4 Statistical analysis

R software (version 4.5) was used for all statistical analyses. The continuous variables in this cohort, such as age, BMI, and treatment cycles, are summarized as M(Q1-Q3) and analyzed using the Mann-Whitney U test instead of the t-test because they departed from normality on testing. Categorical variables are described by counts and percentages; the chi-square test was used to evaluate group differences, and the Fisher exact test was used when anticipated cell counts were less than five.

Multivariable binary logistic regression was performed with CIP occurrence as the dependent variable. With 33 CIP events in this investigation, the maximum number of variables in the model was limited to four when taking into account the restriction of events per variable (13). Based on prior meta-analyses and large cohort studies on core CIP risk factors, prior thoracic radiotherapy, pulmonary comorbidity, and combination therapy were included as established risk factors (9, 10). Age was added as a prespecified covariate. Combination therapy was defined as a composite binary variable, coded as positive if a patient received chemotherapy and/or thoracic radiotherapy concurrently with or after ICI initiation, and negative otherwise. This definition was applied consistently to both CIP and non-CIP patients. Patients receiving both combination chemotherapy and combination radiotherapy were counted once within this composite variable. Effect estimates were presented as odds ratios (ORs) with 95% confidence intervals (CIs). Before interpreting the model, variable independence was assessed using the variance inflation factor (VIF). The receiver operating characteristic curve (ROC)-derived area under the curve (AUC) was used to evaluate model discrimination, and the Hosmer–Lemeshow goodness-of-fit test was used to evaluate calibration.

Patients with CIP were further divided into a mild group (grades 1-2) and a moderate-to-severe group (grade ≥3) according to CTCAE grade. Exploratory univariate comparisons of basic information, disease features, treatment regimens, and clinical outcomes were performed between the two groups. Because the number of moderate-to-severe CIP events was small, Firth penalized logistic regression was used to analyze factors associated with CIP severity. This approach was selected to reduce small-sample bias and complete separation in model estimation. Based on clinical relevance and event-number restrictions, only pulmonary comorbidity and prior thoracic radiotherapy were included as candidate variables related to baseline lung injury. Throughout, two-sided tests were used, and findings were displayed as OR (95% CI) along with the matching P value. The significance level was set at P<0.05.

To explore the cumulative effect of multiple risk factors on CIP occurrence, the number of coexisting risk factors identified by multivariable logistic regression (range 0-3) was calculated for each patient. This exploratory approach was used to examine whether the co-occurrence of multiple risk factors was associated with a higher risk of CIP, and was not intended as a validated risk-stratification tool.

## 3 Results

### 3.1 Overall occurrence of CIP

**Figure 1:**
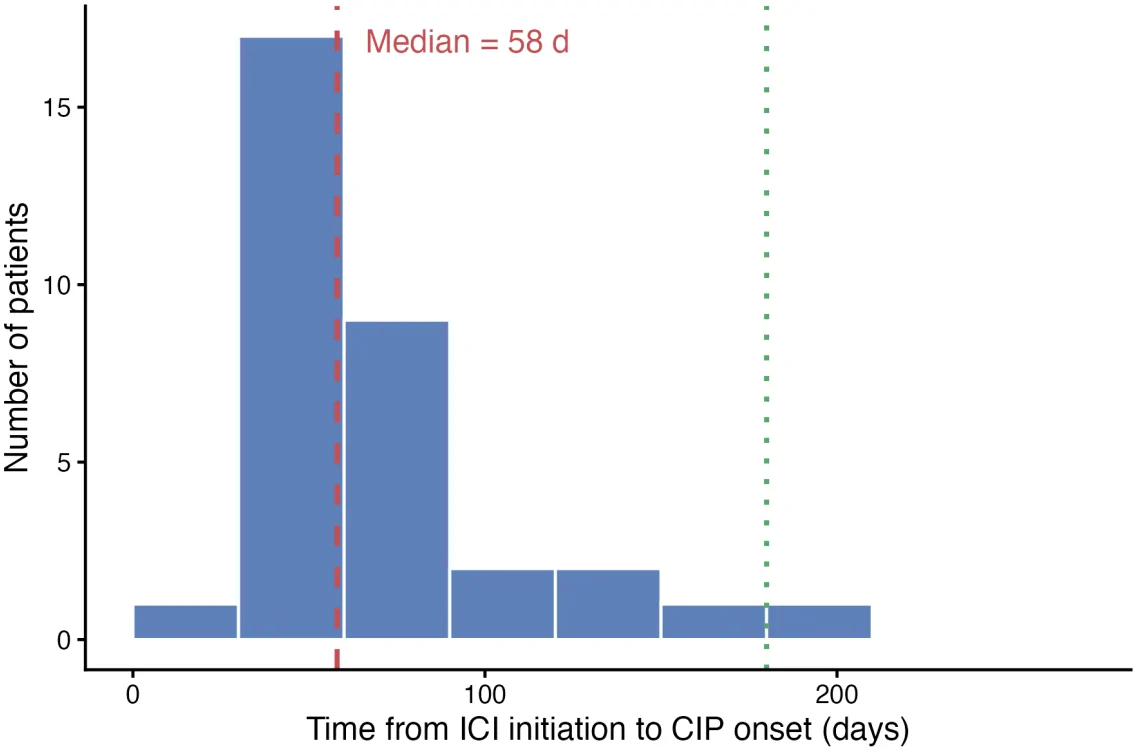
Distribution of CIP onset time (n=33). The red dashed line indicates the median onset time of 58 days, and the green dotted line indicates the 6-month time point.

Among the 206 elderly patients with lung cancer, 33 developed CIP, with an incidence of 16.0%. The median follow-up duration of the whole cohort was 499 days, and 92.7% (191/206) of patients had a follow-up duration of at least 6 months. Among the 33 patients with CIP, the median time to onset was 58 (45-82) days, and 81.8% occurred within 90 days after ICI therapy initiation. CTCAE grading showed that 22 patients (66.7%) had mild CIP (grades 1-2), and 11 patients (33.3%) had moderate-to-severe CIP (grade ≥3). The overall characteristics of CIP are shown in **Table 1**, and the distribution of onset time is shown in **Figure 1**.

**Table 1:** Overall characteristics of 33 patients with CIP.

Item	Category	n	Percentage/value
CIP incidence	–	33	16.0%
Onset time (days), M (Q1–Q3)	–	–	58 (45-82)
Onset time distribution	≤90 days	27	81.8%
	>90 days	6	18.2%
CTCAE grade	Grade 1	8	24.2%
	Grade 2	14	42.4%
	Grade 3	7	21.2%
	Grade 4	4	12.1%
Severity	Mild (grades 1-2)	22	66.7%
	Moderate-to-severe (grade ≥3)	11	33.3%
Corticosteroid use	Yes	22	66.7%
ICI management	Temporary interruption	16	48.5%
	No discontinuation	10	30.3%
	Permanent discontinuation	7	21.2%
Hospital stay (days), M (Q1–Q3)	–	–	9 (8-13)
CIP trajectory	Improved	27	81.8%
	Stable	2	6.1%
	Progression	4	12.1%
All-cause death	–	5	15.2%

Three patients who died during CIP progression are counted in both “progression” and “all-cause death”; two patients died after CIP improvement.

### 3.2 Univariate analysis of CIP occurrence

Pulmonary comorbidity and prior thoracic radiotherapy differed between patients with and without CIP. Patients who developed CIP were more likely to have pulmonary comorbidity (60.6% vs. 30.6%, P<0.05) and prior thoracic radiotherapy (54.5% vs. 29.5%, P<0.05). Among the 33 patients in the CIP group, 27 (81.8%) received combination therapy, including 17 who received combination chemotherapy only, 5 who received combination radiotherapy only, and 5 who received both. Among the 173 patients in the non-CIP group, 110 (63.6%) received combination therapy, including 81 with combination chemotherapy only, 11 with combination radiotherapy only, and 18 with both. The composite combination therapy variable differed significantly between the CIP and non-CIP groups (P = 0.042). See **Table 2**.

**Table 2:** Univariate analysis of CIP occurrence.

Variable	Category	CIP group (n=33)	Non-CIP group (n=173)	P value
Age (years), M (Q1–Q3)	–	73.0 (72.0-76.0)	72.0 (70.0-75.0)	0.157
BMI (kg/m^2^), M (Q1–Q3)	–	22.7 (21.3-26.3)	23.0 (21.4-24.9)	0.596
Treatment cycles, M (Q1–Q3)	–	8.0 (5.0-10.0)	8.0 (6.0-10.0)	0.595
Sex, n (%)	Male	26 (78.8)	120 (69.4)	0.377
	Female	7 (21.2)	53 (30.6)	
Smoking history, n (%)	Yes	22 (66.7)	84 (48.6)	0.086
	No	11 (33.3)	89 (51.4)	
ECOG score, n (%)	0	6 (18.2)	11 (6.4)	0.143
	1	17 (51.5)	95 (54.9)	
	2	9 (27.3)	57 (32.9)	
	3	1 (3.0)	10 (5.8)	
Pathological type, n (%)	Adenocarcinoma	15 (45.5)	97 (56.1)	0.319
	Squamous cell carcinoma	12 (36.4)	52 (30.1)	
	Small-cell lung cancer	6 (18.2)	18 (10.4)	
	Other/unclassified	0 (0.0)	6 (3.5)	
Clinical stage, n (%)	Stage III	7 (21.2)	54 (31.2)	0.345
	Stage IV	26 (78.8)	119 (68.8)	
Pulmonary comorbidity, n (%)	Yes	20 (60.6)	53 (30.6)	0.002
	No	13 (39.4)	120 (69.4)	
ICI type, n (%)	PD-1 inhibitor	18 (54.5)	125 (72.3)	0.069
	PD-L1 inhibitor	15 (45.5)	48 (27.7)	
Prior thoracic radiotherapy, n (%)	Yes	18 (54.5)	51 (29.5)	0.009
	No	15 (45.5)	122 (70.5)	
Combination chemotherapy, n (%)	Yes	22 (66.7)	99 (57.2)	0.414
	No	11 (33.3)	74 (42.8)	
Combination radiotherapy, n (%)	Yes	10 (30.3)	29 (16.8)	0.115
	No	23 (69.7)	144 (83.2)	
Combination therapy, n (%)	Yes	27 (81.8)	110 (63.6)	0.042
	No	6 (18.2)	63 (36.4)	

### 3.3 Multivariable analysis of CIP occurrence

**Figure 2:**
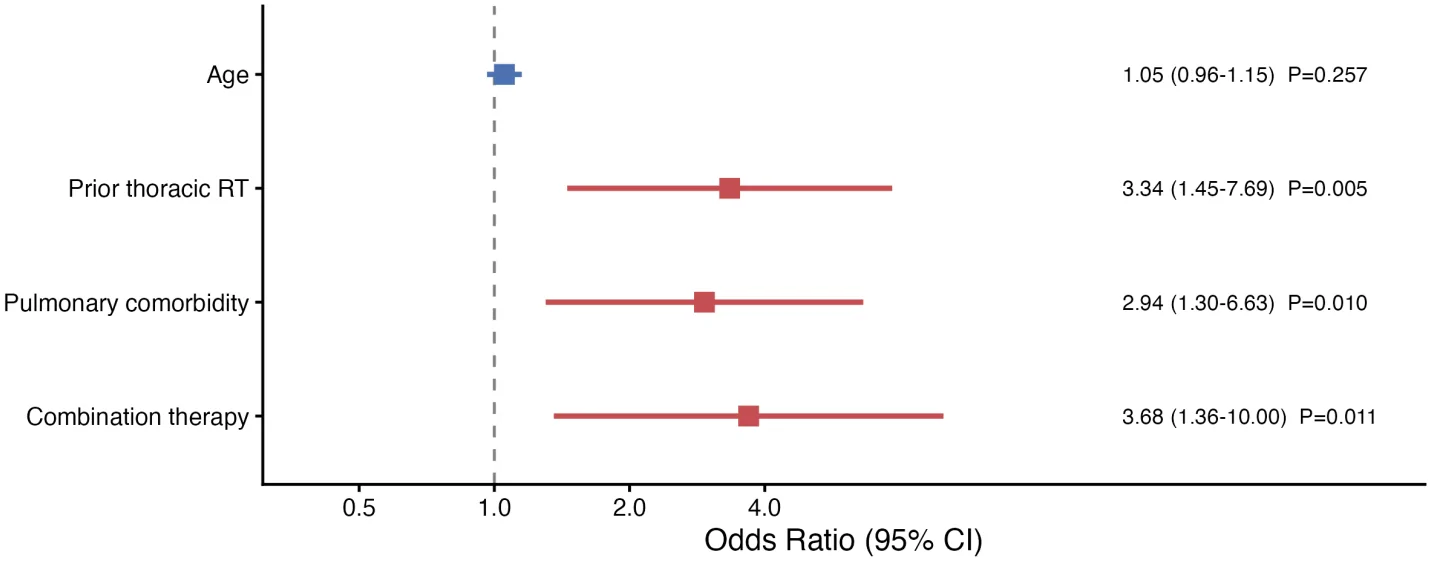
Forest plot of independent risk factors for CIP occurrence. Squares indicate OR values, and horizontal lines represent 95% CIs.

Multivariable logistic regression showed that prior thoracic radiotherapy, pulmonary comorbidity, and combination therapy were independently associated with CIP occurrence. Age was not a significant predictor of CIP (P = 0.257). See **Table 3** and **Figure 2**.

**Table 3:** Multivariable logistic regression for CIP occurrence.

Variable	β	OR	95% CI	P value
Age	0.052	1.05	0.96-1.15	0.257
Prior thoracic radiotherapy	1.206	3.34	1.45-7.69	0.005
Pulmonary comorbidity	1.077	2.94	1.30-6.63	0.010
Combination therapy	1.304	3.68	1.36-10.00	0.011

Combination therapy was a composite variable defined as ICI combined with chemotherapy and/or radiotherapy. VIF values were approximately 1. There were 33 CIP events.

**Figure 3:**
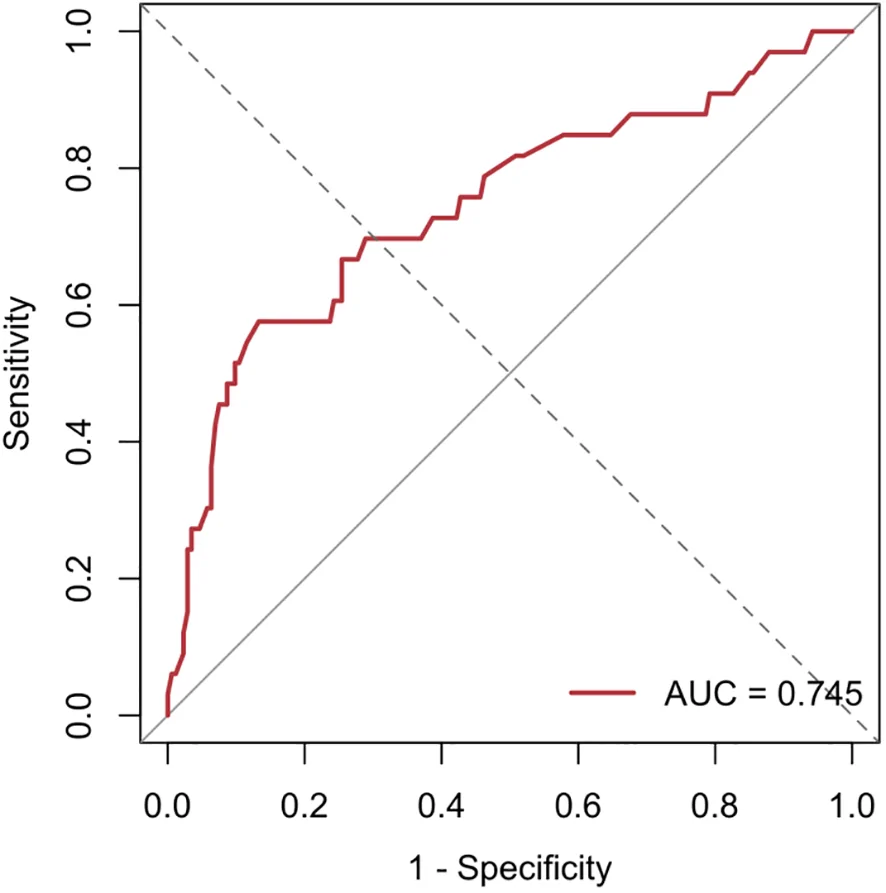
ROC curve of the multivariable model for predicting CIP occurrence.

ROC curve analysis showed that the AUC of this model for predicting CIP occurrence was 0.745, indicating moderate discrimination. The Hosmer-Lemeshow test showed acceptable calibration (χ^2^ = 9.093, P = 0.334). See **Figure 3**.

### 3.4 Factors associated with CIP severity

To analyze factors related to CIP severity, the 33 patients with CIP were divided into a mild group (n=22) and a moderate-to-severe group (n=11) according to CTCAE grade. Univariate analysis showed that the proportion of pulmonary comorbidity, the proportion of prior thoracic radiotherapy, and pathological type distribution differed between the two groups (all P<0.05). See **Table 4**.

**Table 4:** Comparison between patients with mild and moderate-to-severe CIP.

Variable/indicator	Category	Mild group (n=22)	Moderate-to-severe group (n=11)	P value
Age (years), M (Q1–Q3)	–	73.0 (70.5-77.5)	72.0 (72.0-75.5)	0.578
BMI (kg/m^2^), M (Q1–Q3), M (Q1–Q3)	–	23.1 (21.5-26.1)	22.0 (20.9-27.0)	0.954
Treatment cycles, M (Q1–Q3)	–	6.5 (5.0-9.8)	9.0 (7.0-10.0)	0.299
Sex, n (%)	Male	18 (81.8)	8 (72.7)	0.661
	Female	4 (18.2)	3 (27.3)	
Smoking history, n (%)	Yes	16 (72.7)	6 (54.5)	0.437
	No	6 (27.3)	5 (45.5)	
ECOG score, n (%)	0	3 (13.6)	3 (27.3)	0.713
	1	12 (54.5)	5 (45.5)	
	2	6 (27.3)	3 (27.3)	
	3	1 (4.5)	0 (0.0)	
Pathological type, n (%)	Adenocarcinoma	14 (63.6)	1 (9.1)	0.010
	Squamous cell carcinoma	6 (27.3)	6 (54.5)	
	Small-cell lung cancer	2 (9.1)	4 (36.4)	
Clinical stage, n (%)	Stage III	6 (27.3)	1 (9.1)	0.378
	Stage IV	16 (72.7)	10 (90.9)	
Pulmonary comorbidity, n (%)	Yes	10 (45.5)	10 (90.9)	0.022
	No	12 (54.5)	1 (9.1)	
ICI type, n (%)	PD-1 inhibitor	11 (50.0)	7 (63.6)	0.712
	PD-L1 inhibitor	11 (50.0)	4 (36.4)	
Prior thoracic radiotherapy, n (%)	Yes	9 (40.9)	9 (81.8)	0.034
	No	13 (59.1)	2 (18.2)	
Combination chemotherapy, n (%)	Yes	14 (63.6)	8 (72.7)	0.709
	No	8 (36.4)	3 (27.3)	
Combination radiotherapy, n (%)	Yes	6 (27.3)	4 (36.4)	0.696
	No	16 (72.7)	7 (63.6)	

**Figure 4:**
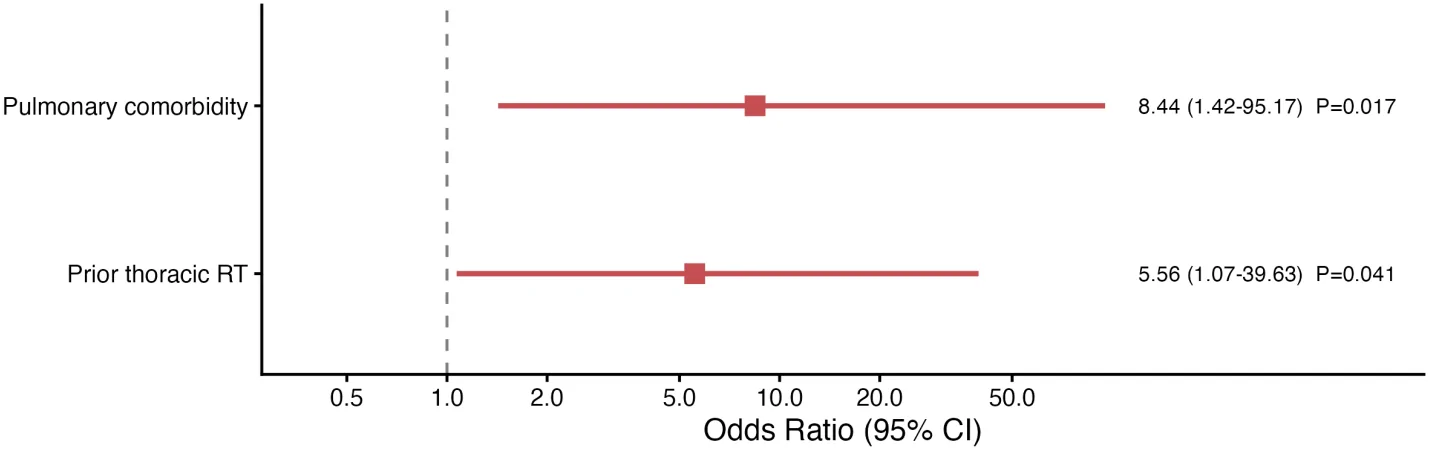
Forest plot of risk factors associated with CIP severity.

Firth penalized logistic regression was performed to identify factors related to CIP severity. Pulmonary comorbidity and prior thoracic radiotherapy showed a trend toward association with moderate-to-severe CIP. See **Table 5** and **Figure 4**.

**Table 5:** Firth penalized logistic regression analysis.

Variable	OR	95% CI	P value
Pulmonary comorbidity	8.44	1.42–95.17	0.017
Prior thoracic radiotherapy	5.56	1.07–39.63	0.041

The outcome variable was moderate-to-severe CIP (CTCAE grade ≥3).

### 3.5 Clinical management and outcomes according to CIP severity

Patients with moderate-to-severe CIP required more intensive clinical management than those with mild CIP. They had a longer hospital stay, were more likely to receive corticosteroids, and more frequently underwent permanent ICI discontinuation (all P<0.01). Clinical outcomes were also poorer in this group: all four cases of CIP progression and all five deaths during follow-up occurred exclusively in the moderate-to-severe group (CIP trajectory, P = 0.008; all-cause death, P = 0.002). See **Table 6**.

**Table 6:** Clinical management and outcomes according to CIP severity.

Indicator	Mild group (n=22)	Moderate-to-severe group (n=11)	P value
Hospital stay (days), M (Q1–Q3)	8.0 (7.0-9.0)	15.0 (13.5-17.0)	<0.001
Corticosteroid use, n (%)	11 (50.0)	11 (100.0)	0.005
ICI management, n (%)			
Temporary interruption	12 (54.5)	4 (36.4)	<0.001
No discontinuation	10 (45.5)	0 (0.0)	
Permanent discontinuation	0 (0.0)	7 (63.6)	
CIP trajectory, n (%)			0.008
Improved	20 (90.9)	7 (63.6)	
Stable	2 (9.1)	0 (0.0)	
Progression	0 (0.0)	4 (36.4)	
All-cause death, n (%)	0 (0.0)	5 (45.5)	0.002

Three patients who died during CIP progression are counted in both “progression” and “all-cause death”; two patients died after CIP improvement.

### 3.6 Association between the number of coexisting risk factors and CIP occurrence

The incidence of CIP increased significantly with a greater number of coexisting risk factors (P<0.001), suggesting that the co-occurrence of multiple risk factors may be associated with a higher risk of CIP. This analysis is exploratory and does not represent a validated risk-stratification tool. See **Table 7**.

**Table 7:** CIP incidence according to the number of coexisting risk factors.

Number of coexisting risk factors	Total number	CIP cases	CIP incidence
0 factors	21	1	4.8%
1 factors	111	9	8.1%
2 factors	54	13	24.1%
3 factors	20	10	50.0%
Trend test	–	–	OR=3.21 (95% CI: 1.94-5.31), P<0.001

## 4 Discussion

CIP is one of the most common and potentially fatal immune-related adverse events in ICI treatment, and is particularly clinically challenging in the elderly lung cancer population (14). Based on real-world data from 206 elderly patients with lung cancer who received ICI treatment, this study systematically analyzed the clinical characteristics of CIP occurrence, risk factors, severity-related factors, and clinical outcomes. The study found that the incidence of CIP was 16.0%, and 81.8% of CIP cases occurred within 90 days after ICI treatment, with mild cases predominating. Multivariate logistic regression analysis showed that prior thoracic radiotherapy, underlying lung disease, and combination therapy were related to the occurrence of CIP. Further severity analysis found that underlying lung disease and previous history of chest radiotherapy were associated with moderate to severe CIP; the hospitalization time of patients with moderate to severe CIP was significantly longer, and the glucocorticoid use rate and ICI permanent discontinuation rate were significantly higher. The above results suggest that for elderly patients undergoing ICI treatment for lung cancer, CIP management should not only focus on post-occurrence treatment, but also pay attention to the identification of relevant risk factors, symptom monitoring and timely assessment in the early stages of treatment.

This study’s 16.0% incidence of CIP was within the range found in earlier research on ICI-treated lung cancer patients (9). Compared to patients with other malignancies, individuals with lung cancer had a higher risk of developing CIP. This may be related to baseline lung lesions, smoking exposure, prior thoracic treatment, and higher sensitivity of lung tissue to immune-mediated inflammatory injury (9, 15). The subjects included in this study were all elderly patients with lung cancer, a population generally recognized to have reduced pulmonary reserve and a high prevalence of chronic lung disease. This underscores the clinical relevance of CIP risk in this group; however, as this study did not include a younger comparator group, it cannot establish whether the identified risk factors are specific to elderly patients or would also apply across other age groups. The median time to CIP onset was 58 days, and most occur within 3 months after treatment, suggesting that early ICI treatment is a critical window for CIP monitoring. For nursing work, the first 3 months after the start of treatment should be regarded as a key observation period, especially new or worsening cough, shortness of breath, chest tightness, decreased activity tolerance, hypoxemia and imaging changes.

Prior thoracic radiotherapy remained an independent predictor of CIP in this study. Chest radiotherapy can cause alveolar epithelial cell damage, local inflammatory reaction, increased capillary permeability, and pulmonary interstitial fibrosis changes (16). Even after radiotherapy is completed, chronic inflammatory microenvironment and structural damage may still exist in lung tissue (16). When patients receive ICI treatment, the immune system is reactivated, and areas of lung damage caused by previous radiotherapy may be more susceptible to immune-mediated inflammatory reactions, thereby increasing the risk of CIP (7). In this study, the risk of CIP in patients with a history of chest radiotherapy was approximately 3.34 times that of patients without a history of radiotherapy, suggesting that a history of chest radiotherapy should be an important part of the risk assessment before ICI treatment, which is in line with Qin et al.’s findings (17).

Pulmonary comorbidity disease is also an important independent risk factor for the development of CIP. This study shows that patients with underlying lung disease are at a significantly increased risk of developing CIP. Structural lung disorders leave the lung with less functional margin: chronic inflammation persists, gas exchange is impaired, and the parenchymal architecture is already partly disrupted before any immune insult. Emphysema, prior tuberculosis, interstitial lung disease, and pulmonary fibrosis all fit this pattern (18). When ICI induces immune-related lung injury, such patients are more likely to develop clinical symptoms due to insufficient lung functional reserve and progress to more severe pneumonia manifestations (19). In the exploratory severity analysis, underlying lung disease showed a possible association with moderate-to-severe CIP, suggesting that it may not only affect whether CIP occurs, but also its severity. However, this finding is based on a small number of events (n=11) and requires confirmation in larger cohorts. Therefore, for elderly lung cancer patients with underlying lung diseases, nursing assessment should not only focus on the recording level of “presence or absence of underlying diseases”, but should further focus on the type of underlying lung disease, symptom level, blood oxygenation status, previous lung function and basic changes in imaging.

Combination therapy was also independently associated with CIP. Due to prior research and biological evidence supporting its link with CIP, it was included in the multivariable model despite not being significant in the univariate analysis (10), after adjusting for other factors, the combination therapy was independently associated with the occurrence of CIP, suggesting that its effect may be reflected after controlling for confounding factors. In this study, patients receiving combination therapy were at increased risk of CIP. ICI combined with chemotherapy or radiotherapy may increase the risk of adverse pulmonary reactions through multiple mechanisms: chemotherapy drugs can cause toxic damage to lung tissue, radiotherapy can cause local lung damage, and ICI can enhance the immune inflammatory response (8). The stacking of multiple treatments may make lung tissue more susceptible to inflammation amplification. Elderly patients who receive combined therapy, especially those who also have a history of chest radiotherapy or underlying lung diseases, may warrant closer attention for CIP. Baseline respiratory and pulmonary function assessment should be performed before treatment, and dynamic monitoring should be strengthened during the treatment process.

This study further found that previous chest radiotherapy history, underlying lung disease and combined therapy have an additive effect on the occurrence of CIP: as the number of risk factors increases, the incidence of CIP shows a significant increasing trend. This finding suggests that multiple clinical risk factors may be associated with increased CIP risk, and patients with multiple risk factors may have a higher likelihood of developing CIP (6). Based on this, this study further explored the relationship between the coexistence of multiple risk factors and the risk of CIP. The results showed that as the number of coexisting risk factors increased, the incidence of CIP showed an increasing trend, suggesting that factors such as previous chest radiotherapy history, underlying lung diseases, and combined treatments may have a cumulative effect, jointly affecting the risk of CIP. However, this exploratory analysis of coexisting risk factors was only analyzed based on this study cohort, has not undergone internal or external validation, and cannot be used as a clinical prediction tool. In the future, it is still necessary to further verify the impact of different combinations of risk factors on the risk of CIP through multicenter studies with larger sample sizes.

It is worth noting that age was not shown to be an independent risk factor for the occurrence of CIP in the multifactor model. This may be related to the fact that the subjects included in this study were all elderly patients, and the age distribution range was relatively concentrated, which weakened the statistical effect of age itself. But this does not mean that age is not clinically important. Elderly patients are often accompanied by decreased lung function, increased comorbidities, decreased immune regulatory capacity, and weakened treatment tolerance (20). These factors may indirectly affect the risk of CIP through underlying lung disease, performance status, or treatment selection. Therefore, in nursing management, age should still be used as an important background factor in comprehensive risk assessment, and it is not appropriate to judge the level of risk based on age alone.

The analysis of CIP severity showed that patients with moderate-to-severe CIP had higher proportions of pulmonary comorbidity and prior thoracic radiotherapy. Firth penalized logistic regression showed that underlying pulmonary disease and prior thoracic radiotherapy may be associated with moderate-to-severe CIP. However, due to the limited number of moderate to severe CIP events, the estimates remain highly uncertain, which still need to be further verified in larger sample studies. Still, the direction of the results is clinically plausible. Pulmonary comorbidity and previous thoracic radiotherapy both suggest that the lung has been injured before ICI therapy. When immune-related inflammation occurs, these patients may have less reserve. They may also be more likely to develop hypoxemia, worsening symptoms, and slow recovery (21). In nursing assessment, these two factors should be treated as warning signs for severe CIP.

Additionally, this study discovered that patients with moderate to severe CIP had significantly longer hospital stays, as well as significantly higher rates of glucocorticoid use and permanent ICI withdrawal. All cases of CIP progression and all follow-up deaths also occurred in this group. Given the unadjusted nature of this comparison and the imbalance in pathological type between groups, these findings should be interpreted as descriptive rather than as evidence of a causal or independent association between CIP severity and clinical outcome. They nonetheless suggest that increasing CIP severity is accompanied by greater treatment burden and may warrant closer monitoring for continuity of anti-tumor treatment. Therefore, the focus of CIP management should not be limited to treatment after occurrence, but should move forward to the early risk assessment, early symptom monitoring, and mild stage intervention. Based on the risk factors and severity-related factors identified in this study, nursing practice should pay attention to the early identification and dynamic monitoring of patients with relevant risk factors. In the early stage of ICI treatment, respiratory symptom assessment, blood oxygen monitoring and imaging follow-up should be strengthened, and patients with suspicious symptoms should be promptly clinically evaluated to promote early detection and standardized management of CIP.

Based on the relevant risk factors and clinical outcome characteristics discovered in this study, the following aspects can be focused on in nursing practice. First of all, before ICI treatment, attention should be paid to related factors such as previous chest radiotherapy history, underlying lung diseases, and combined treatments, and the patient’s basic respiratory status and lung condition assessment should be improved. Secondly, in the early stage of ICI treatment, especially within 3 months after treatment, monitoring of related symptoms such as cough, shortness of breath, chest tightness, decreased activity tolerance, and changes in oxygen saturation should be strengthened to promote early identification of CIP. For patients who have developed CIP, symptom observation, treatment management, and complication monitoring should be carried out based on the severity of the disease. The above nursing management measures are mainly based on the findings of this study and previous clinical experience, and their actual effects still need to be further verified through prospective studies.

This study has several limitations. First, it was a single-center retrospective study, and the findings need external validation in other regions and medical institutions. Second, this study mainly analyzed factors related to the occurrence and severity of CIP based on clinical characteristics. Some treatment-related factors, biomarkers and imaging characteristics that may affect the risk of CIP were not included in the analysis, including PD-L1 expression level, specific ICI agents, lines of immunotherapy, radiotherapy-related parameters (radiotherapy dose and time interval with ICI treatment), baseline lung function and baseline interstitial lung abnormalities. These unmeasured factors may lead to residual confounding, and future studies can further incorporate the above variables in larger samples and multi-center cohorts to improve the understanding of factors associated with CIP occurrence and severity and severity-related factors. Third, the number of moderate to severe CIP cases in this study was small (n=11). Although Firth logistic regression was used to reduce the estimation bias caused by small samples and complete separation, the confidence intervals of the estimates results were wide, so the results of the related factor analysis should be interpreted with caution and mainly as exploratory findings. Fourth, because pathological type differed markedly between the mild and moderate-to-severe CIP groups (P = 0.010), the observed differences in clinical outcome between severity groups should not be interpreted as evidence of an independent association between CIP severity and prognosis; the comparison was unadjusted and confounded by underlying tumor histology, treatment continuity, and other unmeasured factors. Finally, the nursing management recommendations proposed in this study based on risk factor analysis and clinical outcomes still lack validation in prospective nursing intervention studies. In the future, multi-center, prospective studies are needed to further evaluate the impact of relevant management measures on the early identification, disease progression and patient treatment continuity of CIP.

## 5 Conclusion

In summary, this study showed that the incidence of CIP in elderly patients with lung cancer receiving ICI therapy was 16.0%. Most cases occurred within 3 months after treatment and were mild. Prior thoracic radiotherapy, pulmonary comorbidity, and combination therapy were independent risk factors for CIP. In exploratory analysis, pulmonary comorbidity and prior thoracic radiotherapy showed a possible association with moderate-to-severe CIP, although this finding requires confirmation in larger cohorts. Patients with moderate-to-severe CIP had longer hospital stays, a higher rate of glucocorticoid use, higher proportions of permanent ICI discontinuation, and all cases of CIP progression and follow-up deaths occurred in this group. Therefore, the results of this study suggest that among elderly patients undergoing ICI treatment for lung cancer, attention should be paid to factors such as a history of thoracic radiotherapy, underlying lung diseases, and combined treatments, and symptom monitoring and clinical evaluation during treatment should be strengthened. The findings of this study can provide reference for early identification and nursing management of CIP.

## Data Availability

The raw data supporting the conclusions of this article will be made available by the authors, without undue reservation.
