# Supplementary material for: Risk factors, severity-related factors, and clinical outcomes of immune checkpoint inhibitor-associated pneumonitis in elderly patients with lung cancer
Source: Front Oncol. 2026 Sep 3;16:1944973. doi: 10.3389/fonc.2026.1944973 (PMC13581803; doi:10.3389/fonc.2026.1944973)
Supplement: Supplementary file 1 [file Table1.docx]

**Table S1. Distribution of individual pulmonary comorbidity components among patients with pulmonary comorbidity (n=73)**

| **Component condition** | **n** | **% of patients with pulmonary comorbidity (n=73)** |
| --- | --- | --- |
| COPD | 46 | 63.0% |
| Emphysema | 22 | 30.1% |
| Pulmonary fibrosis | 16 | 21.9% |
| Interstitial lung disease | 8 | 11.0% |
| Previous pulmonary tuberculosis | 6 | 8.2% |

Note: Percentages sum to more than 100% because patients could have more than one component condition simultaneously (e.g., COPD with pulmonary fibrosis). No patients were classified under the "other chronic structural lung diseases" component of the pulmonary comorbidity definition; all 73 patients were fully accounted for by the five listed conditions.
